# *N*-Aryl-7-hydroxy-5-oxo-2,3-dihydro-1*H*,5*H*-pyrido-[3,2,1-*ij*]quinoline-6-carboxamides. The Synthesis and Effects on Urinary Output

**DOI:** 10.3390/scipharm86020012

**Published:** 2018-04-09

**Authors:** Igor V. Ukrainets, Lyudmila V. Sidorenko, Mykola Y. Golik, Igor M. Chernenok, Lina A. Grinevich, Alexandra A. Davidenko

**Affiliations:** 1 Department of Pharmaceutical Chemistry, National University of Pharmacy, 53 Pushkinska St., 61002 Kharkiv, Ukraine; slv.ludmila@i.ua (L.V.S.); pereval.ukr@gmail.com (I.M.C.); 2 Department of Analytical Chemistry, National University of Pharmacy, 4 Valentynivska St., 61168 Kharkiv, Ukraine; aptekar4009@gmail.com; 3 Department of Medical Chemistry, National University of Pharmacy, 4 Valentynivska St., 61168 Kharkiv, Ukraine; grinevich.lina@gmail.com; 4 Department of Pharmaceutical Chemistry, N. I. Pirogov Vinnitsa National Medical University, 56 Pirogov St., 21018 Vinnitsa, Ukraine; davidenko@vnmu.edu.ua

**Keywords:** anilines, 4-hydroxyquinolin-2(1*H*)-ones, *N*-aryl-7-hydroxy-5-oxo-2,3-dihydro-1*H*,5*H*-pyrido[3,2,1-*ij*]quinoline-6-carboxamides, pyridoquinolines, amidation, diuretic activity

## Abstract

Continuing a targeted search for new leading structures with diuretic action among tricyclic derivatives of hydroxyquinolines, which are of interest as potential inhibitors of aldosterone synthase, the synthesis of a series of the corresponding pyrido[3,2,1-*ij*]quinoline-6-carboxanilides was carried out by amidation of ethyl-7-hydroxy-5-oxo-2,3-dihydro-1*H*,5*H*-pyrido[3,2,1-*ij*]quinoline-6-carboxylate with aniline, aminophenols and *O*-alkylsubstituted analogs with high yields and purity. The optimal conditions of this reaction are proposed; they make it possible to prevent partial destruction of the original heterocyclic ester and thereby avoid formation of specific impurities of 7-hydroxy-2,3-dihydro-1*H*,5*H*-pyrido[3,2,1-*ij*]quinolin-5-one. To confirm the structure of all substances obtained, elemental analysis, nuclear magnetic resonance (NMR) spectroscopy, and mass spectrometry were used. Moreover, the peculiarities of their ^1^H and ^13^C-NMR spectra, as well as their mass spectrometric behavior under conditions of electron impact ionization, were discussed. The effect of pyrido[3,2,1-*ij*]quinoline-6-carboxanilides on the urinary function of the kidneys was studied in white rats of both genders by the standard method of oral administration at a dose of 10 mg/kg. Testing was conducted in comparison with hydrochlorothiazide, as well as with structurally close pyrrolo[3,2,1-*ij*] quinoline-5-carboxanilides studied earlier with the same substituents in the anilide fragments. It was found that addition of one methylene unit to the heterocycle partially hydrogenated and annelated with the quinolone core has a positive impact on biological properties—most of the substances studied exhibit a statistically significant diuretic effect exceeding the activity of not only hydrochlorothiazide, in some cases, but also the action of the structural analogs. The important structural and biological regularities, which are common with pyrroloquinolines and introduced by a chemical modification, were revealed. The importance of the presence in the structure of terminal amide fragments of tricyclic quinoline-3-carboxamides of a 4-methoxy-substituted aromatic ring was particularly marked. The expediency of further study of pyridoquinolines as promising diuretic agents has been shown.

## 1. Introduction

Currently, arterial hypertension is considered to be one of the most globally prevalent and important medical and social problems of mankind. The insidiousness of this disease lies not only in the fact that its manifestations significantly reduce the life quality of patients suffering from high blood pressure [1]; the numerous pathological changes that hypertension slowly but steadily causes in the human body are much worse, leading to the formation of other, often more serious, diseases or complications, and ultimately to a sharp increase in mortality. Therefore, the search for new, safe, and effective agents to treat hypertension is a priority of pharmaceutical and medical chemistry.

Over the last 10–15 years, the leading role in the drug therapy of hypertension, as well as in ischemic heart disease and chronic heart failure, has quite reasonably belonged to diuretics. These drugs are not directly antihypertensive agents. However, they eliminate large amounts of liquid from the body, and thus help to reduce blood pressure to the physiological norm [2,3,4,5]. There is a rather vast range of currently known diuretics, which are drugs of different groups that are significantly different in terms of their mechanism of action, effects on the electrolytic balance, indications for use, side effects, etc. [1,2,3,4,5]. Taking into account the prospect of creating innovative drugs for the treatment of hypertension, diuretics affecting the renin-angiotensin-aldosterone system are probably of greatest interest. The basis for this assumption is the fact that the trigger pathogenetic mechanism of cardiovascular diseases is often a prolonged activation of exactly this system [6,7]. Moreover, it should be emphasized that, by virtue of targeting the individual components of the renin-angiotensin-aldosterone system, diuretics are divided into several subgroups. The first and most numerous of these groups is angiotensin II inhibitors, or AT1-receptor blockers [8]. The second, and more clinically important, subgroup of competitive aldosterone receptor antagonists has long been represented by the first and only drug of this type—Spironolactone [9]. Additionally, only recently, a new (structurally similar) aldosterone receptors blocker—Eplerenone—has appeared on the pharmaceutical market; it possesses higher selectivity in relation to mineralocorticoid receptors, better tolerability and a complete absence of undesirable antiandrogenic effects. As a result, today, this drug is widely and successfully used for the treatment of hypertension and post-infarction heart failure, worldwide [10].

Finally, the third subgroup—inhibitors of aldosterone synthase—is unfortunately not yet represented by drugs officially approved for medical use. However, exactly this direction is considered to be the most promising in creating new agents for the treatment of hypertension, since it has a simple and, at the same time, very original idea: not to block the access of aldosterone to the corresponding receptors or, if necessary, even to stop its production at all [11]. Theoretically, for this purpose it is sufficient to neutralize aldosterone synthase, a key enzyme that catalyzes the final stages of the biosynthesis of aldosterone, at the right time with a suitable chemical agent, and the problem of preventing the adverse effects of this hormone on the body will be solved.

**Figure 1:**
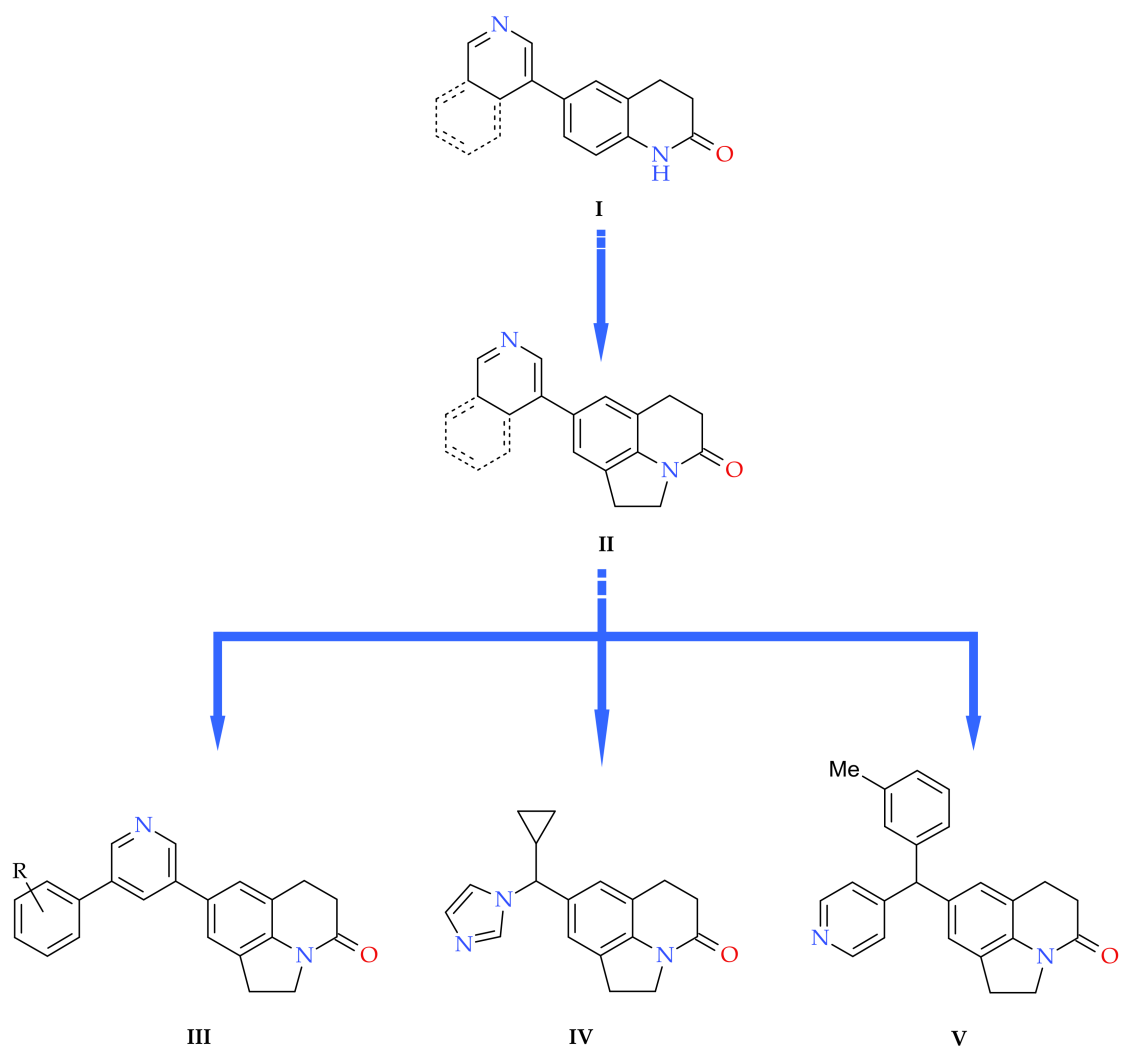
The stages of optimization for synthetic aldosterone synthase inhibitors of quinolone series [18,19,20,21,22,23].

However, it is not so easy to implement this idea in practice; but the high intensity and, most importantly, the effectiveness of studies carried out by many research groups in this field allow us to hope that the creation of a synthetic inhibitor of aldosterone synthase that could be suitable for medical use is only a matter of time. Moreover, derivatives of some aza-heterocycles have shown good results [6,12,13,14,15,16,17]. First of all, our attention was drawn to publications describing quinolin-2-ones **I** [18,19,20], especially their further optimization to tricyclic pyrroloquinolines **II** and then to **III**–**V** (Figure 1) characterized by high selectivity and the possibility of oral administration [21,22,23].

**Figure 2:**
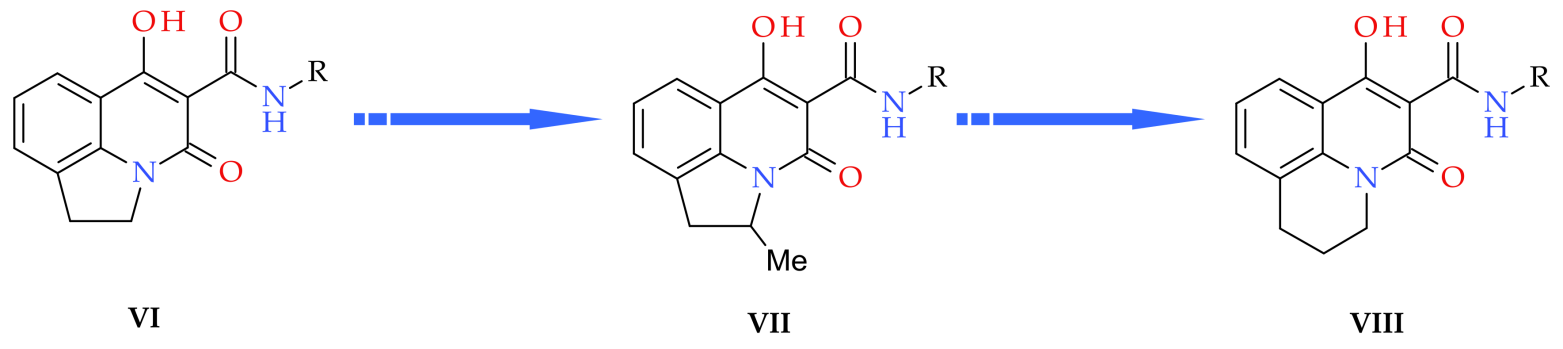
The chemical modification of tricyclic quinolone diuretics from pyrroloquinolines **VI** [24,25] to their 2-methylsubstituted analogs **VII** [26,27] and further to pyridoquinolines **VIII** [28,29].

The structural similarity of these compounds with recently described pyrrolo[3,2,1-*ij*]quinoline-5-carboxanilides **VI** and their 2-methyl-substituted analogs **VII** (Figure 2), which possess a low toxicity, a high diuretic activity, moderate kaliuretic properties, a dehydration effect and a pronounced ability to reduce blood pressure in hypertensive animals [24,25,26,27], suggests that the biological effects of the group of substances studied are also, to some extent, due to the mechanism of inhibition of aldosterone production. 

Therefore, in the future, compounds like amides **VI** and **VII** deserve more attention and further study as potential inhibitors of aldosterone synthase. Until then—at the stage of finding decent structure leaders—a group of their close pyridoquinoline analogs **VIII** differing only in one additional methylene link in the cycle annelated with the quinolone core was synthesized. Thus, this report seeks to answer the question of what effect this modification will have on diuretic properties.

## 2. Materials and Methods

### 2.1. Chemistry

^1^Н- and ^13^С-NMR spectra were acquired on a Varian Mercury-400 (Varian Inc., Palo Alto, CA, USA) instrument (400 and 100 MHz, respectively) in dimethyl sulfoxide-*d*_6_ (DMSO-*d*_6_) with tetramethylsilane as internal standard. The chemical shift values were recorded on a *δ* scale and the coupling constants (*J*) in hertz. The following abbreviations were used in reporting spectra: s = singlet, d = doublet, t = triplet, q = quartet, quin = quintet, m = multiplet. The electron impact mass spectra (EI-MS) were recorded on a Varian 1200 L (Varian Inc., Walnut Creek, CA, USA) mass spectrometer with complete scanning in the *m*/*z* range from 35 to 700 and direct sample inlet. The electron impact ionization was at 70 eV. Elemental analysis was performed on a Euro Vector EA-3000 (Eurovector SPA, Redavalle, Italy) microanalyzer. Melting points were determined in a capillary using a Electrothermal IA9100X1 (Bibby Scientific Limited, Stone, UK) digital melting point apparatus. The synthesis of starting ethyl 7-hydroxy-5-oxo-2,3-dihydro-1*H*,5*H*-pyrido[3,2,1-*ij*]-quinoline-6-carboxylate (**1**) was carried out by the method in the study [28].

### 2.2. General Procedure for the Synthesis of 7-Hydroxy-5-oxo-2,3-dihydro-1H,5H-pyrido[3,2,1-*ij*]quinoline-6-carboxanilides (***2a***–***h***)

A mixture of ethyl ester **1** (2.73 g, 0.01 mol), and the corresponding aniline (0.01 mol) was stirred and allow to stand at 130–140 °C for 5–15 min. The reaction mixture was then cooled to about 100 °C, ethanol (10–15 mL) was carefully added, and thoroughly triturated. The precipitated corresponding anilide **2a**–**h** was filtered off, washed with cold alcohol, dried, and recrystallized from the mixture of *N*,*N*-dimethylformamide (DMF) and ethanol (1:3). Anilides **2a**–**h** were colorless or white with yellowish crystals.

*N-Phenyl-7-hydroxy-5-oxo-2,3-dihydro-1H,5H-pyrido[3,2,1-ij]quinoline-6-carboxamide* (**2a**). Yield: 96%; m.p. 178–180 °C, 179 °C [30], 177–178 °C [31]; ^1^H-NMR (400 MHz, DMSO-*d*_6_): δ 16.50 (s, 1H, 7-OH), 12.64 (s, 1H, NH), 7.97 (d, 1H, *J* = 8.0, H-8), 7.66 (d, 1Н, *J* = 8.3, Н-2′,6′), 7.44 (d, 1H, *J* = 7.5, H-10), 7.33 (t, 1Н, *J* = 7.9, Н-3′,5′), 7.17 (t, 1H, *J* = 7.6, H-9), 7.10 (t, 1Н, *J* = 7.5, H-4′), 4.13 (t, 2H, *J* = 5.8, СН_2_-3), 3.00 (t, 2H, *J* = 6.0, СН_2_-1), 2.12 (quin, 2H, *J* = 5.8, СН_2_-2). ^13^C-NMR (100 MHz, DMSO-*d*_6_): δ 173.7 (7-С-ОH), 167.4 (CONHAr), 159.0 (5-C=O), 146.2, 140.4, 133.0, 129.1 (3′,5′-C), 125.5, 124.3, 122.8, 121.9, 121.3 (2′,6′-C), 116.5, 95.6 (6-C), 41.2 (3-CH_2_), 26.6 (1-CH_2_), 19.5 (2-CH_2_). EI-MS (*m*/*z*, %): 320 (78) [M]^+^, 227 (24), 93 (100). Analytically calculated (Anal. Calcd.) for C_19_H_16_N_2_O_3_: C, 71.24; H, 5.03; N, 8.74%. Found: C, 71.35; H, 4.94; N, 8.67%.

*N-(2-Hydroxyphenyl)-7-hydroxy-5-oxo-2,3-dihydro-1H,5H-pyrido[3,2,1-ij]quinoline-6-carboxamide* (**2b**). Yield: 88%; m.p. 281–283 °C; ^1^H-NMR (400 MHz, DMSO-*d*_6_): δ 16.84 (s, 1H, 7-OH), 12.59 (s, 1H, NH), 9.73 (s, 1H, 2′-OH), 8.24 (d, 1Н, *J* = 8.3, Н-6′), 7.97 (d, 1H, *J* = 8.0, H-8), 7.42 (d, 1H, *J* = 7.4, H-10), 7.16 (t, 1H, *J* = 7.7, H-9), 6.92–6.07 (m, 2Н, Н-3′,5′), 6.76 (t, 1Н, *J* = 6.4, Н-4′), 4.16 (t, 2H, *J* = 5.8, СН_2_-3), 3.00 (t, 2H, *J* = 6.0, СН_2_-1), 2.12 (quin, 2H, *J* = 5.8, СН_2_-2). ^13^C-NMR (100 MHz, DMSO-*d*_6_): δ 170.9 (7-С-ОH), 168.7 (CONHAr), 160.8 (5-C=O), 153.0 (2′-C-OH), 147.3, 136.2, 130.9, 125.9, 124.4, 122.3, 121.6, 120.9, 119.0, 114.8, 114.2, 96.2 (6-C), 41.4 (3-CH_2_), 26.9 (1-CH_2_), 19.9 (2-CH_2_). EI-MS (*m*/*z*, %): 336 (81) [M]^+^, 227 (28), 109 (100). Anal. Calcd. for C_19_H_16_N_2_O_4_: C, 67.85; H, 4.79; N, 8.33%. Found: C, 67.92; H, 4.86; N, 8.27%.

*N-(3-Hydroxyphenyl)-7-hydroxy-5-oxo-2,3-dihydro-1H,5H-pyrido[3,2,1-ij]quinoline-6-carboxamide* (**2c**). Yield: 93%; m.p. 192–194 °C; ^1^H-NMR (400 MHz, DMSO-*d*_6_): δ 16.57 (s, 1H, 7-OH), 12.58 (s, 1H, NH), 9.25 (s, 1H, 3′-OH), 7.96 (d, 1H, *J* = 8.0, H-8), 7.50 (d, 1H, *J* = 7.4, H-10), 7.23 (t, 1H, *J* = 7.7, H-9), 7.19 (s, 1H, Н-2′), 7.10 (t, 1Н, *J* = 8.2, Н-5′), 6.96 (d, 1Н, *J* = 8.0, Н-6′), 6.54 (d, 1Н, *J* = 7.8, Н-4′), 4.14 (t, 2H, *J* = 5.8, СН_2_-3), 3.01 (t, 2H, *J* = 6.1, СН_2_-1), 2.10 (quin, 2H, *J* = 5.8, СН_2_-2). ^13^C-NMR (100 MHz, DMSO-*d*_6_): 170.8 (7-С-ОH), 168.6 (CONHAr), 161.4 (5-C=O), 157.9 (3′-C-OH), 143.2, 136.1, 132.7, 129.6, 125.9, 122.2, 121.4, 120.3, 117.0, 116.1, 112.4, 96.3 (6-C), 41.5 (3-CH_2_), 26.4 (1-CH_2_), 19.6 (2-CH_2_). EI-MS (*m*/*z*, %): 336 (87) [M]^+^, 227 (35), 109 (100). Anal. Calcd. for C_19_H_16_N_2_O_4_: C, 67.85; H, 4.79; N, 8.33%. Found: C, 67.93; H, 4.85; N, 8.37%.

*N-(4-Hydroxyphenyl)-7-hydroxy-5-oxo-2,3-dihydro-1H,5H-pyrido[3,2,1-ij]quinoline-6-carboxamide* (**2d**). Yield: 97%; m.p. 208–210 °C, 208 °C [30]; ^1^H-NMR (400 MHz, DMSO-*d*_6_): δ 16.87 (s, 1H, 7-OH), 12.36 (s, 1H, NH), 8.85 (s, 1H, 4′-OH), 7.97 (d, 1H, *J* = 8.1, H-8), 7.45 (d, 2Н, *J* = 8.6, Н-2′,6′), 7.39 (d, 1H, *J* = 7.4, H-10), 7.14 (t, 1H, *J* = 7.7, H-9), 6.73 (d,2Н, *J* = 8.6, Н-3′,5′), 4.14 (t, 2H, *J* = 5.8, СН_2_-3), 2.99 (t, 2H, *J* = 6.0, СН_2_-1), 2.13 (quin, 2H, *J* = 5.8, СН_2_-2). ^13^C-NMR (100 MHz, DMSO-*d*_6_): δ 170.5 (7-С-ОH), 167.9 (CONHAr), 160.9 (5-C=O), 154.5 (4′-C-OH), 135.8, 132.8, 128.3, 125.2, 122.3 (3′,5′-C), 121.9, 121.7, 115.3 (2′,6′-C), 114.8, 95.2 (6-C), 41.4 (3-CH_2_), 26.9 (1-CH_2_), 19.9 (2-CH_2_). EI-MS (*m*/*z*, %): 336 (88) [M]^+^, 227 (39), 109 (100). Anal. Calcd. for C_19_H_16_N_2_O_4_: C, 67.85; H, 4.79; N, 8.33%. Found: C, 67.80; H, 4.82; N, 8.26%.

*N-(2-Methoxyphenyl)-7-hydroxy-5-oxo-2,3-dihydro-1H,5H-pyrido[3,2,1-ij]quinoline-6-carboxamide* (**2e**). Yield: 84%; m.p. 223–225 °C; ^1^H-NMR (400 MHz, DMSO-*d*_6_): δ 16.69 (s, 1H, 7-OH), 12.73 (s, 1H, NH), 8.37 (d, 1Н, *J* = 8.0, Н-6′), 7.98 (d, 1H, *J* = 8.0, H-8), 7.43 (d, 1H, *J* = 7.3, H-10), 7.16 (t, 1H, *J* = 7.7, H-9), 7.05 (t, 1Н, *J* = 7.6, Н-5′), 6.96 (d, 1Н, *J* = 8.2, Н-3′), 6.92 (t, 1Н, *J* = 7.6, Н-4′), 4.17 (t, 2H, *J* = 5.7, СН_2_-3), 3.98 (s, 3H, OMe), 3.01 (t, 2H, *J* = 6.0, СН_2_-1), 2.14 (quin, 2H, *J* = 5.7, СН_2_-2). ^13^C-NMR (100 MHz, DMSO-*d*_6_): δ 170.3 (7-С-ОH), 164.6 (CONHAr), 161.5 (5-C=O), 152.2 (2′-C-OMe), 150.0, 145.1, 139.0, 136.5, 133.1, 127.2, 122.3, 121.1, 115.4, 114.0, 111.5, 95.8 (6-C), 56.1 (OCH_3_), 41.8 (3-CH_2_), 26.9 (1-CH_2_), 20.0 (2-CH_2_). EI-MS (*m*/*z*, %): 350 (73) [M]^+^, 227 (22), 123 (100). Anal. Calcd. for C_20_H_18_N_2_O_4_: C, 68.56; H, 5.18; N, 8.00%. Found: C, 68.45; H, 5.07; N, 7.92%.

*N-(3-Methoxyphenyl)-7-hydroxy-5-oxo-2,3-dihydro-1H,5H-pyrido[3,2,1-ij]quinoline-6-carboxamide* (**2f**). Yield: 94%; m.p. 176–178 °C, 175 °C [30]; ^1^H-NMR (400 MHz, DMSO-*d*_6_): δ 16.48 (s, 1H, 7-OH), 12.66 (s, 1H, NH), 7.98 (d, 1H, *J* = 8.0, H-8), 7.45 (d, 1H, *J* = 7.3, Н-10), 7.36 (s, 1Н, Н-2′), 7.21 (t, 1H, *J* = 8.1, H-5′), 7.17 (t, 1H, *J* = 7.7, H-9), 7.11 (d, 1H, *J* = 8.1, Н-6′), 6.65 (d, 1H, *J* = 8.2, Н-4′), 4.15 (t, 2H, *J* = 5.8, СН_2_-3), 3.84 (s, 3H, OMe), 3.00 (t, 2H, *J* = 6.0, СН_2_-1), 2.13 (quin, 2H, *J* = 5.8, СН_2_-2). ^13^C-NMR (100 MHz, DMSO-*d*_6_): δ 170.6 (7-С-ОH), 168.4 (CONHAr), 166.6 (3′-C-OMe), 160.1 (5-C=O), 149.1, 146.3, 137.4, 136.3, 132.7, 129.1, 125.2, 121.7, 113.6, 111.3, 107.6, 95.9 (6-C), 54.8 (OCH_3_), 41.3 (3-CH_2_), 26.3 (1-CH_2_), 19.6 (2-CH_2_). EI-MS (*m*/*z*, %): 350 (75) [M]^+^, 227 (27), 123 (100). Anal. Calcd. for C_20_H_18_N_2_O_4_: C, 68.56; H, 5.18; N, 8.00%. Found: C, 68.47; H, 5.11; N, 8.09%. 

*N-(4-Methoxyphenyl)-7-hydroxy-5-oxo-2,3-dihydro-1H,5H-pyrido[3,2,1-ij]quinoline-6-carboxamide* (**2g**). Yield: 97%; m.p. 180–182 °C; ^1^H-NMR (400 MHz, DMSO-*d*_6_): δ 16.65 (s, 1H, 7-OH), 12.48 (s, 1H, NH), 7.97 (d, 1H, *J* = 8.0, H-8), 7.58 (d, 2Н, *J* = 8.9, Н-2′,6′), 7.44 (d, 1H, *J* = 7.4, H-10), 7.17 (t, 1H, *J* = 7.6, H-9), 6.86 (d, 2Н, *J* = 8.9, Н-3′,5′), 4.14 (t, 2H, *J* = 5.8, СН_2_-3), 3.79 (s, 3H, OMe), 3.00 (t, 2H, *J* = 6.0, СН_2_-1), 2.13 (quin, 2H, *J* = 5.8, СН_2_-2). ^13^C-NMR (100 MHz, DMSO-*d*_6_): δ 172.6 (7-С-ОH), 165.4 (CONHAr), 159.1 (5-C=O), 156.4 (4′-C-OMe), 146.1, 134.0, 132.3, 126.4, 123.4, 122.5 (3′,5′-C), 120.0, 118.9, 114.6 (2′,6′-C), 96.2 (6-C), 57.8 (OCH_3_), 41.6 (3-CH_2_), 26.7 (1-CH_2_), 19.8 (2-CH_2_). EI-MS (*m*/*z*, %): 350 (91) [M]^+^, 227 (38), 123 (100). Anal. Calcd. for C_20_H_18_N_2_O_4_: C, 68.56; H, 5.18; N, 8.00%. Found: C, 68.61; H, 5.26; N, 7.94%. 

*N-(4-Ethoxyphenyl)-7-hydroxy-5-oxo-2,3-dihydro-1H,5H-pyrido[3,2,1-ij]quinoline-6-carboxamide* (**2h**). Yield: 92%; m.p. 189–191 °C; ^1^H-NMR (400 MHz, DMSO-*d*_6_): δ 16.70 (s, 1H, 7-OH), 12.47 (s, 1H, NH), 7.97 (d, 1H, *J* = 8.0, H-8), 7.56 (d, 2Н, *J* = 8.8, Н-2′,6′), 7.43 (d, 1H, *J* = 7.4, H-10), 7.18 (t, 1H, *J* = 7.7, H-9), 6.83 (d, 2Н, *J* = 8.8, Н-3′, 5′), 4.14 (t, 2H, *J* = 5.8, СН_2_-3), 4.02 (q, 2H, *J* = 7.0, OСН_2_), 3.01 (t, 2H, *J* = 6.0, СН_2_-1), 2.13 (quin, 2H, *J* = 5.8, СН_2_-2), 1.41 (t, 2H, *J* = 7.0, OСН_2_CH_3_). ^13^C-NMR (100 MHz, DMSO-*d*_6_): δ 173.2 (7-С-ОH), 169.4 (CONHAr), 162.2 (5-C=O), 155.1 (4′-C-OEt), 148.0, 139.7, 137.1, 126.9, 124.1, 121.4 (3′,5′-C), 120.5, 118.3, 116.8 (2′,6′-C), 95.2 (6-C), 63.7 (OCH_2_), 41.8 (3-CH_2_), 26.1 (1-CH_2_), 19.7 (2-CH_2_), 14.8 (CH_3_). EI-MS (*m*/*z*, %): 364 (79) [M]^+^, 227 (34), 137 (100); Anal. Calcd. for C_21_H_20_N_2_O_4_: C, 69.22; H, 5.53; N, 7.69%. Found: C, 69.15; H, 5.45; N, 7.78%.

### 2.3. Pharmacology

#### Diuretic Test

The effect of the synthesized *N*-aryl-7-hydroxy-5-oxo-2,3-dihydro-1*H*,5*H*-pyrido[3,2,1-*ij*]-quinoline-6-carboxamides on the excretory function of the kidneys was studied in white outbred rats of both genders with weights of 180–200 g by the standard method [32]. All experimental animals were given a water load calculated at 25 mL/kg via a gastric tube. The control group was given only the similar amount of water with Tween-80. The tested anilides **2a**–**h** were introduced per os in the form of a thin water suspension stabilized by Tween-80. Then the animals were placed in “metabolic cages”. The primary screening was carried out at a dose of 10 mg/kg, which corresponds to the ED_50_ of one of the most active 6-hydroxy-4-oxo-2,4-dihydro-1*H*-pyrrolo[3,2,1-*ij*]quinoline-5-carboxanilides (**VI**, 2-H, R’ = -C_6_H_4_-OMe-*p*). The values of excretion were registered after 4 h and compared with the control, as well as a known diuretic, hydrochlorothiazide [33], used at its effective dose of 40 mg/kg. Ten experimental animals were involved to obtain statistically reliable results (the significance level of the confidence interval accepted in this work was *p* ≤ 0.05) in testing each of anilides **2a**–**h**, the reference drugs and control.

All biological experiments were carried out in full accord with the European convention on the protection of vertebrate animals used for experimental and other scientific purposes and the Ukrainian Law No. 3447-IV “On protection of animals from severe treatment” (2006) (project ID 1674U13 approved 22 September 2012).

## 3. Results and Discussion

### 3.1. Chemistry

**Scheme 1:**
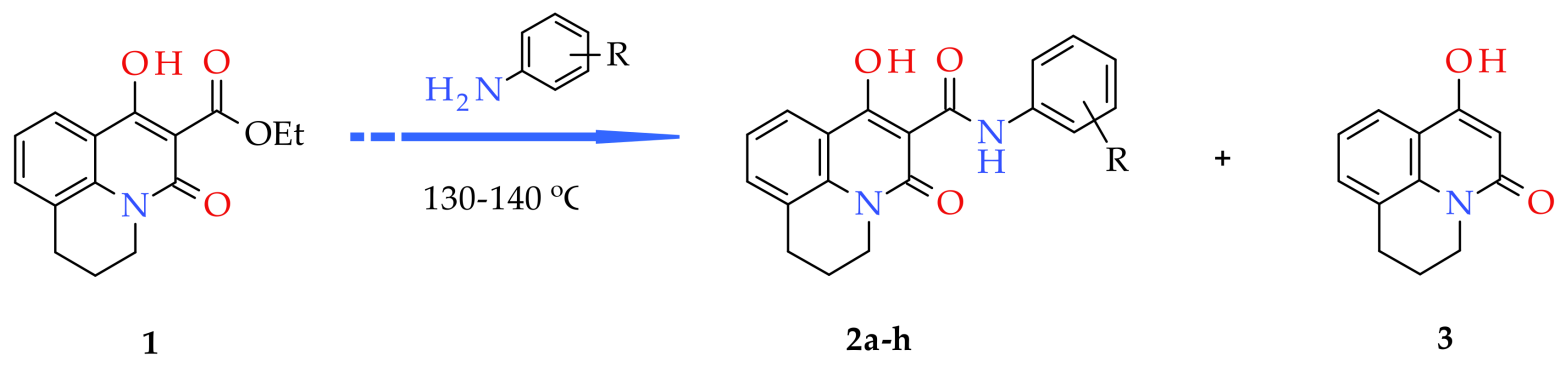
Synthesis of *N*-aryl-7-hydroxy-5-oxo-2,3-dihydro-1*H*,5*H*-pyrido[3,2,1-*ij*]-quinoline-6-carboxamides **2a**–**h** from ethyl ester **1** and anilines.

As is known, the reactivity of the lower alkyl-4-hydroxy-2-oxo-1,2-dihydroquinoline-3-carboxylates remains high, regardless of the structure of the benzene moiety of the molecule and the nature of the substituents at the nitrogen atom [34]. Their tricyclic pyridoquinoline analog **1** is no exception in this respect; it easily reacts with the primary and even secondary anilines or hetaryl amines with formation of the corresponding amides at the temperature of 130–140 °С [28,29]. Unlike the older formulation [30], this simple method (Scheme 1) allows the final compounds **2a**–**h** to be obtained with high yields and purity for a few minutes at the equimolar ratio of reagents; moreover, a high-boiling solvent, as a rule, is not required (see Materials and Methods). However, it should be remembered that heating of the reaction mixture above 140 °C is not desirable, since due to the residual water, which is present in anilines, this process may be accompanied by the partial destruction of the ester groups of the original ester **1** with formation of the secondary 7-hydroxy-2,3-dihydro-1*H*,5*H*-pyrido[3,2,1-*ij*]quinolin-5-one (**3**) as an impurity.

All *N*-aryl-7-hydroxy-5-oxo-2,3-dihydro-1*H*,5*H*-pyrido[3,2,1-*ij*]quinoline-6-carboxamides (**2a**–**h**) synthesized in such a way are colorless or white crystalline substances with a yellowish tint and a narrow interval of melting points (see Materials and Methods). At room temperature, they are moderately soluble in DMF and dimethyl sulfoxide, sparingly soluble in ethanol, and practically insoluble in diethyl ether, hexane and water.

**Figure 3:**
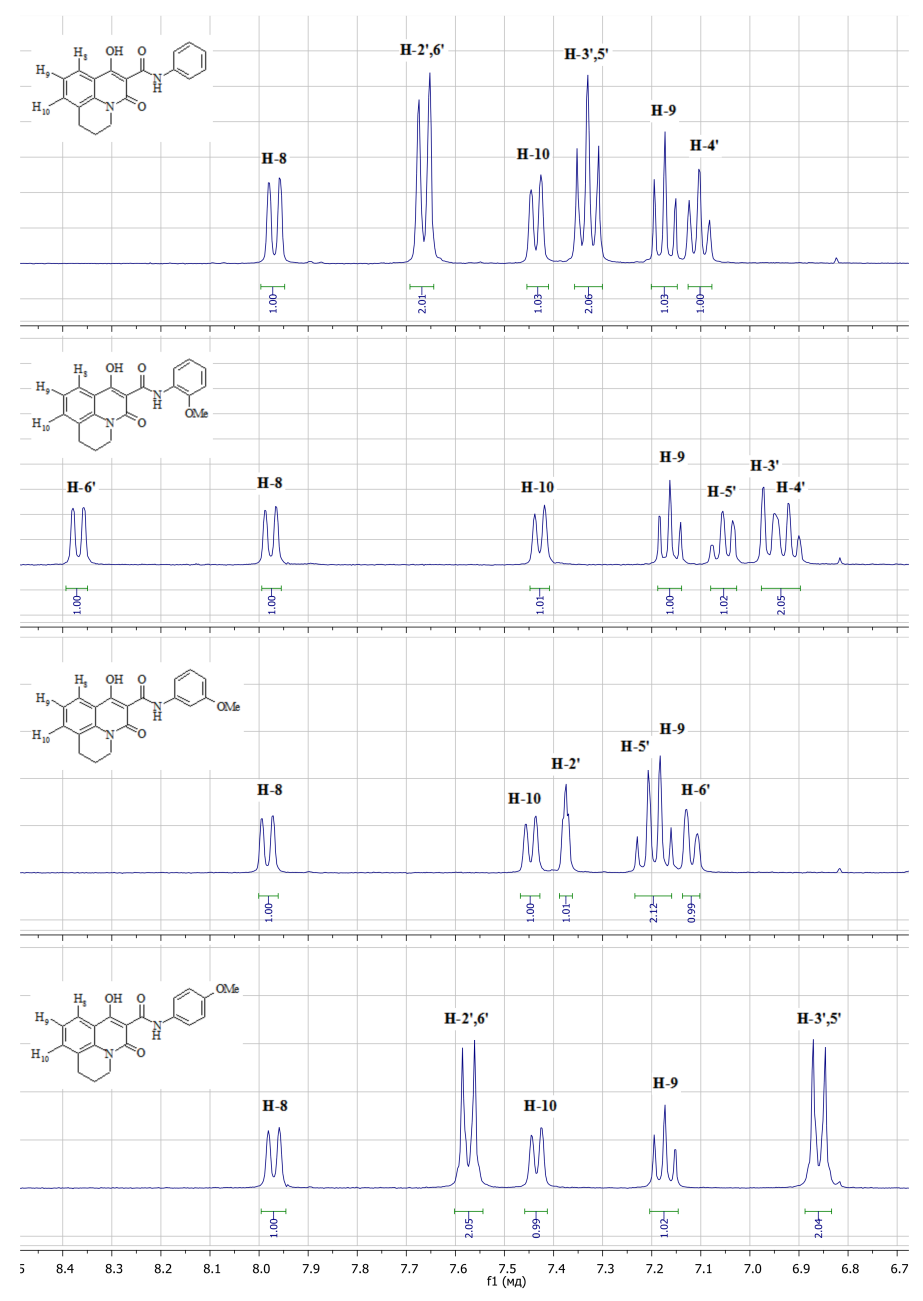
Fragments of ^1^H-NMR spectra (the signals of aromatic protons) of the unsubstituted anilide **2a** and of its monomethoxy-substituted derivatives **2e**–**2g**.

To confirm their chemical structure, elemental analysis, NMR spectroscopy (^1^Н and ^13^С), and mass spectrometry were used. Interpretation of ^1^H NMR spectra of anilides **2a**–**h** does not cause any difficulties. Even in the aromatic region of the spectrum, where there are simultaneously signals of 7–8 protons in the area of less than 1.4 ppm, coincidences of the resonance frequencies are not observed. The signals of aromatic protons of the pyridoquinoline core are typical for this chemical environment; they consist of two doublets (H-8 and H-10) and a triplet (H-9). In ^1^Н-NMR spectra, they remain in the same positions regardless of the substituents in the anilide fragment of the molecule (Figure 3).

Because of the relatively low solubility of *N*-aryl-7-hydroxy-5-oxo-2,3-dihydro-1*H*,5*H*-pyrido[3,2,1-*ij*]quinoline-6-carboxamides (**2a**–**h**) in DMSO-*d*_6_ at room temperature, it is unfortunately not always possible to register their ^13^С-NMR spectra with high quality. Nevertheless, this method allows us to unambiguously confirm the presence of the tetrahydropyridine cycle, alkoxy- and carbonyl groups, and other functional fragments in the compounds studied (see Materials and Methods).

**Scheme 2:**
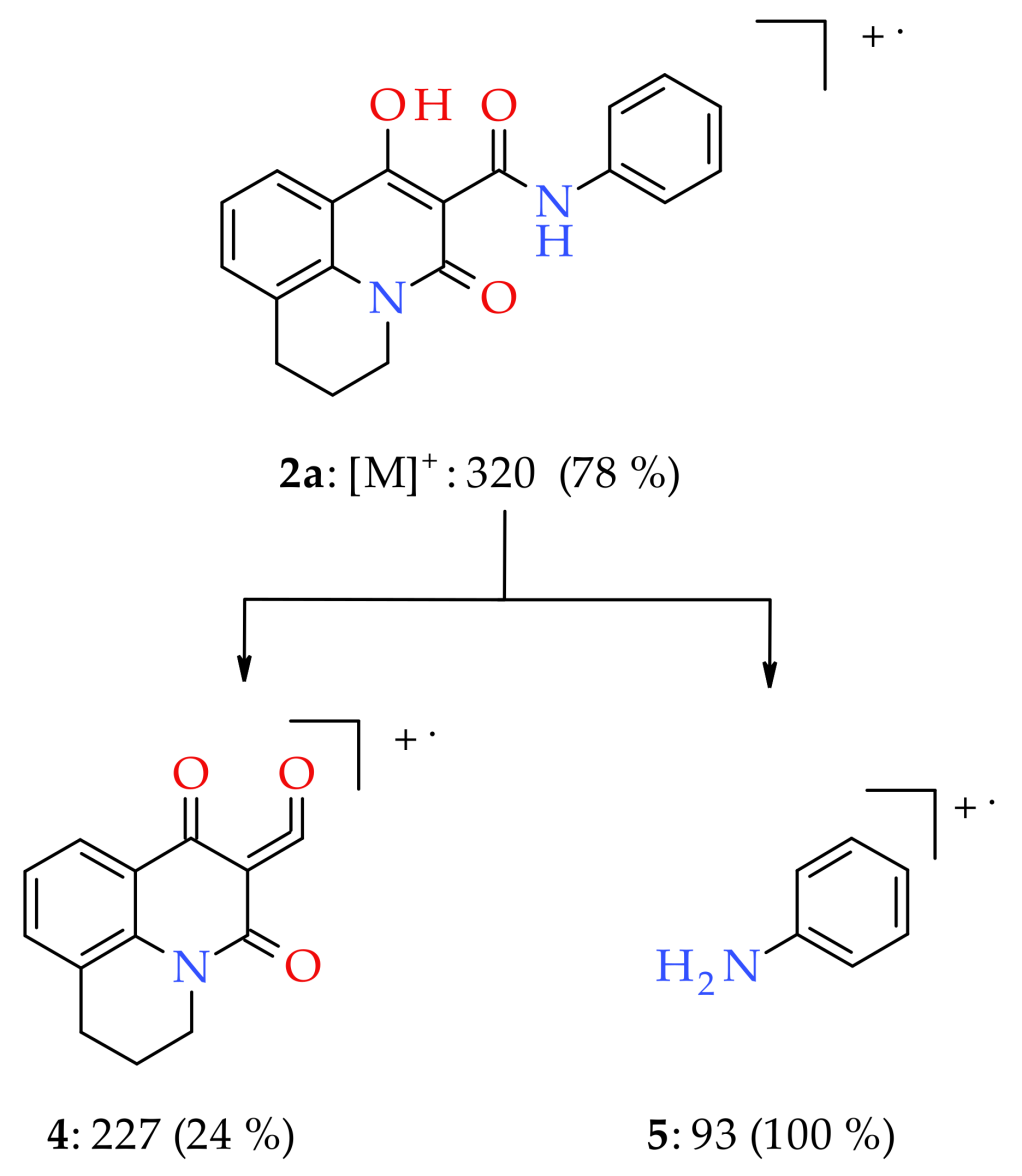
The primary fragmentation of the molecular ion of anilide **2a**.

The analysis of mass spectra registered under conditions of the electron impact ionization shows that anilides **2a**–**h** are very stable compounds and form high-intensity peaks of molecular ions that allow the determination of the molecular weight of the substances tested. The primary fragmentation of molecular ions is shown in the example of anilide **2a**, it proceeds by the ketene type characteristic for 4-hydroxy-2-oxo-1,2-dihydroquinoline-3-carboxamides and is accompanied by the breaking of the acyclic amide bond with formation of tricyclic ketene **4**, which is common to all samples, with *m*/*z* 227 and the corresponding aniline **5** (Scheme 2) giving the most powerful peak in the spectrum with 100% intensity.

### 3.2. Evaluation of Diuretic Activity

The results of the experimental study of the effect of all *N*-aryl-7-hydroxy-5-oxo-2,3-dihydro-1*H*,5*H*-pyrido[3,2,1-*ij*]quinoline-6-carboxamides (**2a**–**h**) synthesized on the urinary function of the kidneys are presented in Table 1, together with the indicators of anilides of 6-hydroxy-4-oxo-2,4-dihydro-1*H*-pyrrolo[3,2,1-*ij*]quinoline-5-carboxylic (**VI**) and 6-hydroxy-2-methyl-4-oxo-2,4-dihydro-1*H*-pyrrolo[3,2,1-*ij*]quinoline-5-carboxylic (**VII**) acids previously tested in similar conditions with the same substituents in the arylamide fragments. In the comparative analysis of these data, the attention was immediately drawn to the pronounced diuretic effect of the unsubstituted anilide **2а** on the background of the moderately intense antidiuretic effect of its pyrroloquinoline analogs **VI** and **VII**. 3-Hydroxy- and 2-methoxy groups significantly changed the biological properties of the substances studied for the better—anilides **2c** and **2е** by their diuretic activity already exceeded not only the structural analogs **VI** and **VII**, respectively, but also hydrochlorothiazide, and in a much lower dose. The position of the methoxy group in the anilide moiety of the molecule interestingly affected the activity. If its gradual increase was previously observed while moving the specified substituent from the *ortho*- to the *meta*- and further to the *para*-position, then there was no direct dependence with transition to pyridoquinolines: the initially high diuretic effect of *ortho*-isomer **2e** was immediately lost in *meta*-analog **2f**, but then again, it recovered to the previous level in *para*-derivative **2g**.

The structural and biological regularities common to all types of the compounds studied were also noted. Thus, in particular, it was found that as in the case of pyrroloquinolines **VI** and **VII**, *N-*(4-methoxyphenyl)-7-hydroxy-5-oxo-2,3-dihydro-1*H*,5*H*-pyrido[3,2,1-*ij*]quinoline-6-carboxamide (**2g**) appeared to be the most active of the whole group. In other words, another experimental confirmation of a positive effect on the diuretic properties of tricyclic quinolinecarboxamides was obtained. This was precisely due to the presence of the 4-methoxysubstituted aromatic ring in the structure of their terminal amide fragments. However, the 4-ethoxy group in all examples considered had a stable, but quite opposite effect. Its presence almost completely deprived the molecule of any significant effect on the urinary function of the kidneys, no matter which cycle was annelated with the hydroquinolone core on its edges *i* and *j*—tetrahydropyridine (anilide **2h**), trihydropyrrole (**VI**) or 2-methyltrihydropyrrole (**VII**) (see Table 1). Therefore, the addition of at least one methylene link in the *О*-methyl substituent as an option for possible optimization of quinoline diuretics is unreasonable. However, this observation does not exclude the possibility of appearance and even enhancement of the diuretic activity in derivatives with longer *O*-alkyl chains.

## 4. Conclusions

This report presents a series of new *N*-aryl-7-hydroxy-5-oxo-2,3-dihydro-1*H*,5*H*-pyrido[3,2,1-*ij*]-quinoline-6-carboxamides obtained within the framework of the integrated program on synthesis, studies of the reactivity, structure, chemical and biological properties of 4-hydroxyquinoline-2-one derivatives and structurally related heterocycles. Being close analogs of the pyrroloquinolone diuretics previously described, these compounds, as the objects of study, are of interest as a source of not only important regularities of the “structure-activity” relationship, but probably new leading structures. The chemical structure of all *N*-aryl-7-hydroxy-5-oxo-2,3-dihydro-1*H*,5*H*-pyrido[3,2,1-*ij*]quinoline-6-carboxamides has been reliably confirmed by elemental analysis, NMR (^1^Н and ^13^С) and mass spectra. According to the results of pharmacological tests, among the compounds synthesized, the samples that can actively enhance the urinary function of the kidneys have been identified. Based on these results, the transformation of the trihydropyrrole heterocycle annelated with the hydroxyquinolone core into the tetrahydropyridine heterocycle is recommended as one of the possible variants for optimization of quinoline diuretics.

## Figures and Tables

**Table 1:** The diuretic activity of anilides **2a**–**h**, their corresponding pyrroloquinoline analogs **VI** and **VII**, and Hydrochlorothiazide.

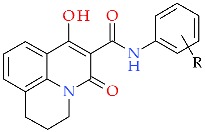				
Entry	Product	R	Diuresis in 4 h	
			mL ^1^	% ^2,3^
1	**2** **a**	H	7.68 ± 0.29	+43 (−21 and −28)
2	**2** **b**	2-OH	4.62 ± 0.22	−14 (− and −12)
3	**2** **c**	3-OH	8.59 ± 0.32	+60 (− and −28)
4	**2** **d**	4-OH	5.91 ± 0.30	+10 (− and −19)
5	**2** **e**	2-OMe	8.70 ± 0.32	+62 (+44 and −58)
6	**2** **f**	3-OMe	6.28 ± 0.35	+17 (+51 and +3)
7	**2** **g**	4-OMe	9.08 ± 0.37	+69 (+126 and +68)
8	**2h**	4-OEt	6.01 ± 0.28	+12 (0 and +6)
9	Hydrochlorothiazide	–	8.11 ± 0.30	+51
10	Control	–	5.37 ± 0.29	–

^1^ All results from biological tests were analyzed statistically using Student’s *t*-test. Effects were regarded as statistically significant at *p ≤* 0.05; ^2^ “+” Indicates increase and “–” inhibition of diuresis when compared with the control taken as 100%; ^3^ The data on the diuretic activity of the corresponding anilides of 6-hydroxy-4-oxo-2,4-dihydro-1*H*-pyrrolo[3,2,1-*ij*]quinoline-5-carboxylic (**VI**) and 6-hydroxy-2-methyl-4-oxo-2,4-dihydro-1*H*-pyrrolo-[3,2,1-*ij*]quinoline-5-carboxylic (**VII**) acids [25,26,27] are given in parentheses.
